# Abscission of flowers and floral organs is closely associated with alkalization of the cytosol in abscission zone cells

**DOI:** 10.1093/jxb/eru483

**Published:** 2014-12-10

**Authors:** Srivignesh Sundaresan, Sonia Philosoph-Hadas, Joseph Riov, Eduard Belausov, Betina Kochanek, Mark L. Tucker, Shimon Meir

**Affiliations:** ^1^Department of Postharvest Science of Fresh Produce, Agricultural Research Organization (ARO), The Volcani Center, Bet-Dagan 5025001, Israel; ^2^The Robert H. Smith Institute of Plant Sciences and Genetics in Agriculture, The Robert H. Smith Faculty of Agriculture, Food and Environment, The Hebrew University of Jerusalem, Rehovot 7610001, Israel; ^3^Department of Ornamental Horticulture, Agricultural Research Organization (ARO), The Volcani Center, Bet-Dagan 5025001, Israel; ^4^Soybean Genomics and Improvement Laboratory, USDA-ARS, Beltsville, MD 20705, USA

**Keywords:** Abscission zone, alkalization, *Arabidopsis* mutants, cytosol, ethylene, flower organs, pH regulation, tomato (*Solanum lycopersicum*), wild rocket (*Diplotaxis tenuifolia*).

## Abstract

*In vivo* changes in the cytosolic pH of abscission zone (AZ) cells were visualized using confocal microscopic detection of the fluorescent pH-sensitive and intracellularly trapped dye, 2’,7’-bis-(2-carboxyethyl)-5(and-6)-carboxyfluorescein (BCECF), driven by its acetoxymethyl ester. A specific and gradual increase in the cytosolic pH of AZ cells was observed during natural abscission of flower organs in *Arabidopsis thaliana* and wild rocket (*Diplotaxis tenuifolia*), and during flower pedicel abscission induced by flower removal in tomato (*Solanum lycopersicum* Mill). The alkalization pattern in the first two species paralleled the acceleration or inhibition of flower organ abscission induced by ethylene or its inhibitor 1-methylcyclopropene (1-MCP), respectively. Similarly, 1-MCP pre-treatment of tomato inflorescence explants abolished the pH increase in AZ cells and pedicel abscission induced by flower removal. Examination of the pH changes in the AZ cells of *Arabidopsis* mutants defective in both ethylene-induced (*ctr1*, *ein2*, *eto4*) and ethylene-independent (*ida*, *nev7*, *dab5*) abscission pathways confirmed these results. The data indicate that the pH changes in the AZ cells are part of both the ethylene-sensitive and -insensitive abscission pathways, and occur concomitantly with the execution of organ abscission. pH can affect enzymatic activities and/or act as a signal for gene expression. Changes in pH during abscission could occur via regulation of transporters in AZ cells, which might affect cytosolic pH. Indeed, four genes associated with pH regulation, vacuolar H^+^-ATPase, putative high-affinity nitrate transporter, and two GTP-binding proteins, were specifically up-regulated in tomato flower AZ following abscission induction, and 1-MCP reduced or abolished the increased expression.

## Highlight

A novel insight is provided into the abscission process, showing for the first time that pH changes in abscission zone cells are involved in execution of floral abscission.

## Introduction

Abscission is a process by which plants shed their organs, such as leaves, flowers, and fruits. Abscission occurs in specialized cells known as the abscission zone (AZ), which develops at the base of the organ to be shed. The AZ is comprised of a few layers of cells that are typically smaller than adjacent cells in the non-AZ (NAZ), and have a denser cytoplasm. The AZ cells are predisposed to respond to abscission signals. Upon induction, these cells secrete cell wall-modifying and hydrolysing enzymes, that loosen the cell wall and degrade the middle lamella between adjacent cells (Sexton and Roberts, 1982; Osborne, 1989; Bleecker and Patterson, 1997; Roberts *et al.*, 2000 2002; Patterson, 2001; Stenvik *et al.*, 2006). In many plant species, the abscission process is induced by ethylene; nonetheless, the rate and degree of abscission depend upon the balance between the levels of auxin and ethylene in the AZ. Thus, the auxin concentration in the AZ must be reduced to render the AZ cells responsive to ethylene (Sexton and Roberts, 1982; Patterson, 2001; Taylor and Whitelaw, 2001; Roberts *et al.*, 2002; Meir *et al*., 2006 2010). Indeed, it was demonstrated that acquisition of ethylene sensitivity in tomato flower AZ correlated with altered expression of auxin-regulated genes evoked by flower removal, which are the source of auxin (Meir *et al.*, 2010).

Although *Arabidopsis* does not abscise its leaves or fruit, its floral organs (petals, sepals, and anthers) do abscise. Over the last two decades, abscission of *Arabidopsis* flower organs has served as a model for abscission research. Recently, by employing different strategies to manipulate auxin levels in the AZs of *Arabidopsis* floral organs, it was shown that auxin signalling is essential for floral organ abscission (Basu *et al.*, 2013). Both ethylene-dependent pathways and an ethylene-independent pathway acted in parallel in *Arabidopsis* floral organ abscission, but were to some degree interdependent. In wild-type (WT) plants, ethylene accelerated the senescence and abscission of floral organs. In ethylene-insensitive mutants, such as *ethylene receptor 1* (*etr1*) and *ethylene-insensitive 2* (*ein2*), abscission was significantly delayed (Bleecker and Patterson, 1997; Patterson, 2001; Butenko *et al*., 2003 2006; Patterson *et al.*, 2003; Patterson and Bleecker, 2004; Chen *et al.*, 2011; Kim *et al.*, 2013*b*). However, although ethylene-insensitive mutants display delayed floral organ abscission, they eventually abscise and exhibit a separation process similar to that of the WT. These observations led to the conclusion that although ethylene accelerates abscission, the perception of ethylene is not essential for floral organ abscission. This indicated that an ethylene-independent pathway exists in *Arabidopsis* floral organ abscission (Bleecker and Patterson, 1997; Patterson *et al.*, 2003; Patterson and Bleecker, 2004).

An ethylene-independent pathway has been characterized for *Arabidopsis* floral organ abscission. This signalling pathway is comprised of several components identified by means of genetic mutations that delayed abscission. A model of the proteins involved in the signal transduction of the ethylene-independent pathway in abscission is presented in the review of Estornell *et al.* (2013). Briefly, *INFLORESENCE DEFICIENT IN ABSCISSION* (*IDA*) (Butenko *et al.*, 2003) encodes a peptide ligand (Stenvik *et al*., 2006 2008) that putatively binds to the redundant receptor-like kinases HAESA (HAE) and HAESA-LIKE2 (HSL2), which activate downstream KNOX-like transcription factors (Cho *et al.*, 2008; Stenvik *et al.*, 2008). Another ethylene-independent mutant is *nevershed* (*nev*) (Liljegren *et al.*, 2009). The *NEVERSHED* (*NEV*) gene encodes an ADP-ribosylation factor-GTPase-activating protein (ARF-GAP) involved in Golgi transport.

Additional genes that affect abscission include the *DELAYED IN ABSCISSION* (*DAB*) genes. Five independent mutants, *dab1*, *2*, *3*, *4*, and *5*, were identified by screening for delayed floral organ abscission (Patterson *et al.*, 2003; Patterson and Bleecker, 2004). While *DAB1*, *2*, and *3* have not been cloned, *DAB4* was found to be allelic to the jasmonic acid co-receptor *CORONATINE INSENSITIVE1* (*COI1*), and its novel allele, *coi1-37* (Kim *et al*., 2013*a*, *b*). Many metabolic and enzymatic processes depend on a specific range of pH, due to regulation of protein structure and function. Various cellular processes are compartmentalized within the organelles, cytosol, and apoplast, each with a distinct function and distinct pH requirements (Casey *et al.*, 2010; Orij *et al.*, 2011; Pittman, 2012). pH has a major role in secretory functions, in which it regulates post-translational modification and sorting of proteins and lipids as they move along the secretory pathway (Paroutis *et al.*, 2004). pH can be a signal and/or a messenger, and changes in pH and H^+^ ions act as a signal for gene expression in various physiological processes (Savchenko *et al.*, 2000; Felle, 2001; Miyara *et al.*, 2010; Orij *et al.*, 2011). Dynamic changes in cytosolic and/or apoplastic pH occur in many plant cell types and in response to stress conditions (Felle, 2001, 2005, 2006; Couldwell *et al.*, 2009; Swanson *et al.*, 2011) and environmental signals, such as pathogen infection (Alkan *et al.*, 2008; Miyara *et al.*, 2010) and gravitropic stimulation (Felle, 2001; Roos *et al.*, 2006). In addition, pH changes can activate several different transporters (Pittman *et al.*, 2005).

Although the possible involvement of pH changes in the abscission process was suggested many years ago by Osborne (1989), no experimental evidence has been provided to support this hypothesis. Osborne proposed that a change in pH occurs during abscission, based on studies in which a decrease in the pH of the cell wall activated cell wall-associated enzymes, such as polygalacturonase (PG), which are considered to operate at a low pH range between 4.5 and 5.5 (Riov, 1974; Ogawa *et al.*, 2009).

Using a pH-sensitive fluorescent indicator, 2’,7’-bis-(2-carboxyethyl)-5(and-6)-carboxyfluorescein-acetoxymethyl (BCECF-AM), an AZ-specific change was observed in the cytosolic pH during abscission, which correlated with both ethylene-dependent and ethylene-independent abscission signalling. Moreover, a strong correlation was demonstrated between pH changes in the AZ cells and execution of organ abscission in three different abscission systems: *A. thaliana*, wild rocket (*Diplotaxis tenuifolia*), and tomato (*Solanum lycopersicum* Mill), and in response to ethylene or its inhibitor, 1 methylcyclopropene (1-MCP). The possible role of pH changes in the abscission process is discussed.

## Materials and methods

### Plant materials and growth conditions

#### Arabidopsis 

*Arabidopsis thaliana* Columbia (Col) WT and mutant lines of the Col ecotype, *constitutive triple response 1* (*ctr1*), *ein2*, *ethylene overproducer 4* (*eto4*), *dab5*, *ida*, and *nev7*, used in this research were generously provided by Dr Sara E. Patterson, University of Wisconsin-Madison, USA. Seeds were surface sterilized for 5min in 1% (v/v) sodium hypochlorite containing 0.05% Triton X-100, followed by five rinses in sterile double-distilled water (DDW). The seeds were placed in Petri dishes with Murashige and Skoog medium (Duchefa Biochemie) containing 2.3g l^–1^ vitamins, 8g l^–1^ plant agar, and 15g l^–1^ sucrose, pH 5.7, and incubated at 4 °C for 4 d in the dark. The dishes were then transferred to a controlled environment room at 24 °C under 16h light, and grown for 10 d before transplanting. The seedlings were transplanted into pots containing Klassman 686 peat:perlite (85:15, v/v) medium with 0.1% (w/v) of a slow release fertilizer (Osmocote, The Scotts Company, Marysville, OH, USA), and covered with Saran polyethylene for 3–5 d, which was then removed. The seedlings were transferred to a controlled growth chamber and grown at 24 °C with supplementary light (100 μmol m^–2^ s^–1^) to maintain a 16h photoperiod until maturity.

#### Wild rocket 

Wild rocket (*D. tenuifolia*) seedlings were grown in 10 litre pots in tuff:peat (50:50, v/v) medium containing 0.1% (w/v) Osmocote slow release fertilizer. Plants were grown under a 30% shade net during July to November.

#### Tomato 

Cherry tomato (*S. lycopersicum*) inflorescences cv. ‘VF-36’ or cv. ‘Shiran’ 1335 (Hazera Genetics Ltd, Israel) were harvested for BCECF fluorescence analyses or microarray experiments (Meir *et al.*, 2010), respectively, from greenhouse-grown plants between 09:00h and 11:00h. Bunches containing at least 2–4 freshly open flowers were brought to the laboratory under high humidity conditions. Closed young flower buds and senesced flowers were removed, and the stem ends were trimmed. Groups of 3–4 bunch explants were placed in vials containing 10ml of 50mg l^–1^ organic chlorine (TOG-6, Gadot Agro, Ltd, Israel) in water to prevent contamination by microorganisms. The vials were divided into two groups: one was incubated at 20 °C after flower removal with a sharp razor blade (control), and the second group was exposed to 1-MCP (0.4 μl l^–1^) in a sealed 200 litre chamber at 20 °C for 2h before flower removal, followed by incubation at 20 °C. Pedicel abscission was monitored in the two groups of explants at various time intervals during a 60h period after flower removal.

### Application of ethylene and 1-MCP, and determination of flower petal abscission in wild rocket

Wild rocket flowering shoots, in which P0–P3 flowers were marked, were exposed to ethylene, 1-MCP, or both. For ethylene treatment, the flowering shoots were placed in vials containing DDW and incubated for 24h under 10 μl l^–1^ ethylene in a 200 litre air-tight chamber at 20 °C. For 1-MCP treatment, the flowering shoots in water were incubated for 2h in 0.4 μl l^–1^ 1-MCP (EthylBloc™, Rohm and Haas, USA) in a 200 litre air-tight chamber at 20 °C. For the combined treatment, the flowering shoots were first exposed for 2h to 1-MCP and then for 22h to ethylene under the same conditions detailed above. After treatment, the flowering shoots were transferred to a controlled observation room maintained at 20±1 °C, 60±10% relative humidity, and a photoperiod of 12h at a light intensity of 14 μmol m^–2^ s^–2^ provided by cool white fluorescent tubes. The rate of flower petal abscission in response to a very delicate finger touch was recorded during incubation until 100% of the petals abscised. Experiments were repeated three times, with 10 flowering shoots each, and analysis of variance (ANOVA) was used for statistical analysis of the data of the three experiments.

### Ethylene production in flowers and siliques at different positions along the inflorescence of *Arabidopsis* Col WT and *ctr1* and *eto4* mutants

*Arabidopsis* plants were grown as described above, and the experiments were conducted when the inflorescences had ~20–23 flowers. Samples of 6–8 whole flowers and/or siliques at specified positions along the inflorescence (P2–P17) of Col (WT) and *ctr1* and *eto4* mutants were excised, weighed, and placed in air-tight sealed 23ml vials that were incubated for 1h at 20 °C under light. Air samples of 3ml were withdrawn from the vials and the ethylene concentration was determined by gas chromatography.

### BCECF fluorescence analyses by confocal microscopy

#### BCECF-AM probe stock and working solutions 

BCECF-AM (Cat-B1150; www.invitrogen.com) was used. A stock solution of the BCECF-AM was dissolved in a high quality anhydrous dimethyl sulphoxide (DMSO) to a final concentration of 10mM. The DMSO stock solution was stored at –20 °C in the dark. The working solution was prepared by adding 1 μl of stock solution to 1ml of phosphate-buffered saline (PBS), pH 7.4, to a final concentration of 10 μM.

#### Sample preparation for microscopic experiments 

*Arabidopsis and wild rocket*. Inflorescences with flowers located at various positions along the inflorescence were harvested ~1h before assaying, placed in DDW, and immediately used for the imaging experiments. Flowers at different developmental stages were excised separately from the inflorescences and placed on microscopic slides. Generally, flower sepals, petals, and stamens were removed using forceps without damaging the carpel, receptacles, and peduncles.

*Tomato.* Samples were collected at specific time points (0, 4, 8, and 14h or 0, 2, 4, and 8h) after flower removal for cross- or longitudinal section images, respectively. Flower AZ (FAZ) tissues were collected from each side of the abscission fracture by excising 3mm thick tissue (proximal and distal) of the AZ and NAZ regions for preparing longitudinal sections. The longitudinal sections were made by cutting down the middle of the tissues with a sharp razor blade, without causing injury, and placing them on microscopic slides. For cross-section preparation, 1mm sections were collected from the middle of the FAZ fracture.

#### Probe loading for microscopic observations 

The BCECF-AM working solution (25 μl for *Arabidopsis* and wild rocket and 10 μl for tomato) was applied onto the surface of the tissue samples, which were then incubated under darkness for 20min. The samples were rinsed four times with PBS to remove excess BCECE-AM. The *Z*-stack images were taken with an Olympus IX-81 confocal laser scanning microscope (CLSM) (FV 500, Olympus Optical Co., Tokyo, Japan), equipped with a 488nm argon-ion laser. Samples were excited by 488nm light and the emission was detected through a BA 505–525 filter. A BA 660 IF emission filter was used to detect chlorophyll autofluorescence. Transmitted light images were obtained using Nomarski differential interference contrast (DIC) microscopy. The relative fluorescence intensity was quantified in the CLSM images using MICA (Multi Image Co-Localization Analysis) software (Cytoview Company, Israel; http://www.cytoview.com/). All experiments were repeated three times with different biological samples from different inflorescences, and representative images are presented.

### Microarray analysis of tomato flower AZ

AZ tissue of tomato flowers was sampled at five time points (0, 2, 4, 8, and 14h) following flower removal, and the pedicel NAZ tissue was sampled at four time points (0, 2, 4, and 14h), with or without 1-MCP pre-treatment as previously described (Meir *et al.*, 2010). RNA extraction and microarray analysis of tomato flower AZ were performed as detailed in Meir *et al.* (2010).

## Results

### A specific increase of cytosolic pH in *Arabidopsis* flower organ AZ cells coincided with floral organ abscission

**Fig. 1.**
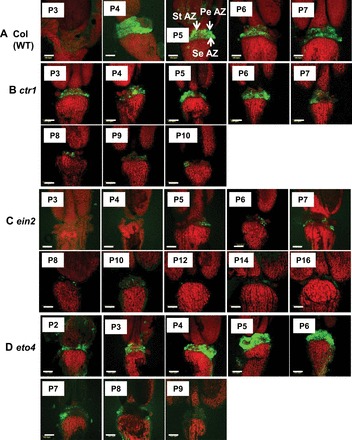
Fluorescence micrographs of BCECF images of flower organ AZ of *Arabidopsis* Col WT (A) and Arabidopsis ethylene-related mutants *ctr1* (B), *ein2* (C), and *eto4* (D), showing pH changes in P3–16 flowers. Intact *Arabidopsis* Col WT and mutant flowers defined according to their position on the inflorescence were sampled separately, incubated in BCECF solution, and examined by CLSM. The microscopic fluorescence images represent merged images of BCECF fluorescence with chlorophyll autofluorescence and bright field images. The increase in pH is shown by green fluorescence, which is distinguished from the red chlorophyll autofluorescence. The arrows in the P5 panel in the first row indicate the location of the flower organ AZ, based on Patterson (2001). PeAZ, petal AZ; StAZ, stamen AZ; SeAZ, sepal AZ. Scale bars=100 μm. The images presented for each plant type (WT or mutant) and positions are representative images out of 3–6 replicates. P1 represents a flower with petals that are first visible (not shown) and P3 represents a fully open flower.

A specific occurrence of BCECF green fluorescence in the cytoplasm of *Arabidopsis* flower organ AZ cells, indicating an increased pH, was observed by confocal microscopy. The increased green fluorescence in the WT occurred mainly in P4 flowers, declined in P5–P7 flowers (Fig. 1A), and was barely detectable in P8 flowers (data not shown). A magnified BCECF image of a P5 flower (Supplementary Fig. S1A, B available at *JXB* online) showed that the green fluorescence was located in the cytosol. This observation was further confirmed by the magnified BCECF image of a cross-section of tomato flower pedicel AZ cells (Supplementary Fig. S1C), showing a strong specific green fluorescence in the cytosol of the AZ cells. In WT flowers, the petals of P6 flowers abscised in response to a very slight touch, while those of P7 and P8 flowers had already abscised (Supplementary Fig. S2). Thus, activation of abscission occurred in P4 and P5 flowers, which is consistent with earlier reports showing that the abscission process in *Arabidopsis* WT, expressed in decreased petal break strength, is initiated in P4 flowers (González-Carranza *et al.*, 2002; Patterson and Bleecker 2004; Butenko *et al.*, 2006; Basu *et al.*, 2013). Based on the pattern of increased fluorescence in the cytosol of AZ cells (Fig. 1A), it is likely that the increase in pH coincides with the abscission processes in *Arabidopsis* flowers.

To correlate further the pH changes in the AZ cells with flower organ abscission, the changes in the BCECF fluorescence were examined in several *Arabidopsis* mutants displaying different flower abscission phenotypes. Three ethylene-related mutants, *ctr1*, *ein2*, and *eto4*, as well as three ethylene-independent mutants, *ida*, *nev7*, and *dab5*, were used. In *ctr1*, the green fluorescence intensity was already high in P3 flowers and remained relatively high up to P7 flowers, in which the fluorescence began to decline (Fig. 1B). The *ctr1* mutant showed an early abscission of petals and sepals starting in P5 flowers, while the stamen remained attached even in P9 flowers (Supplementary Fig. S3 at *JXB* onlline). In *ein2*, a delayed abscission mutant, the BCECF fluorescence intensity was very low or barely detected in P3–P16 flowers (Fig. 1C) as compared with the WT (Fig. 1A). Flower organ abscission in *ein2* occurred in P10–P14 flowers (data not shown), similar to previously reported data for this mutant (Patterson and Bleecker, 2004; Chen *et al.*, 2011). However, it is important to emphasize that the abscission process in the ethylene-insensitive mutants, *ein2* and *etr1*, started in P6 flowers and proceeded gradually until completion in P14 flowers, as evidenced by the decrease in petal break strength (Patterson and Bleecker, 2004). Therefore, the gradual decrease in petal break strength in *ein2* (Patterson and Bleecker, 2004) correlated well with the low but prolonged BCECF fluorescence intensity detected in P5–P10 flowers (Fig. 1C). Conversely, in the ethylene-overproducing mutant, *eto4*, the BCECF fluorescence started to increase in P2 flowers, peaked in P5 and P6 flowers, and declined between P7 and P9 flowers (Fig. 1D). In *eto4*, the abscission rate was significantly faster, and all the floral organs were already abscised in P5 flowers (Supplementary Fig. S4). Thus, the results of the ethylene-related *Arabidopsis* mutants support the correlation between floral organ abscission and alkalization of the cytosol (Supplementary Figs S3, S4).

**Fig. 2.**
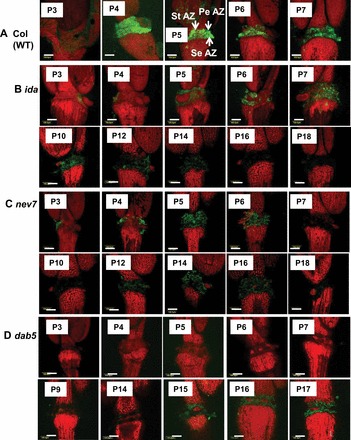
Fluorescence micrographs of BCECF images of flower organ AZ of *Arabidopsis* Col WT (A) and *Arabidopsis* abscission-related mutants *ida* (B), *nev7* (C), and *dab5* (D), showing pH changes in P3–P18 flowers. PeAZ, petal AZ; StAZ, stamen AZ; SeAZ, sepal AZ. Scale bars=100 μm. The experiment was performed as detailed in Fig. 1. The images presented for each plant type (WT or mutant) and flower position are representative images out of 3–6 replicates.

BCECF fluorescence intensity in the floral organ AZ of the ethylene-independent mutants, *ida* (Fig. 2B) and *nev7* (Fig. 2C), and in the delayed abscission mutant *dab5* (Fig. 2D) was very low as compared with the WT (Fig. 2A). The *ida* mutant is characterized by a decrease in petal break strength from P6 to P10 flowers, followed by an increase from P12 to P20 flowers (V-shape pattern) (Butenko *et al.*, 2003; Stenvik *et al.*, 2008; Liu *et al.*, 2013). This V-shape pattern could be seen in *ida* plants, as the P10 flower petals abscised during handling in the BCECF fluorescence experiments. No abscission was observed along the inflorescence of *ida* (data not shown), which is consistent with previous reports (Butenko *et al.*, 2003; Stenvik *et al.*, 2008). Although the BCECF fluorescence in *ida* was low, a low intensity fluorescence could be observed in P5–P14 flowers (Fig. 2B), which coincided with the gradual decrease in petal break strength in P5–P10 flowers. Similar to *ida*, no abscission was observed along the inflorescence of *nev7* (data not shown), which is consistent with previous reports (Liljegren *et al.*, 2009; Liu *et al.*, 2013). The *nev7* mutant is also characterized by a V-shape pattern in petal break strength. However, the decrease in break strength is very moderate and the lowest value is detected in P6 flowers (Liu *et al.*, 2013). The fluorescence intensity in P3–P18 flowers was very low (Fig. 2C) compared with the WT (Fig. 2A). Yet, some fluorescence was observed in P3–P6 flowers (Fig. 2C) that correlated with the moderate decrease in petal break strength in these flower positions (Liu *et al.*, 2013). It should be noted that in *dab5* no BCECF fluorescence could be observed in P3–P14 flowers (Fig, 2D). The BCECF fluorescence was detected only in P15–P17 flowers (Fig. 2D), when organ separation was first observed (Supplementary Fig. S5 at *JXB* online), which is consistent with previous observations (S.E. Patterson and A.B. Bleecker, unpublished data). Similar to the ethylene-insensitive mutants, *ein2* and *etr1*, a gradual decrease in petal break strength occurred in *dab5*, starting from P8 flowers until the completion of abscission (S.E. Patterson, personal communication). This decrease in petal break strength from P12 flowers until the completion of abscission was less significant than in the WT, and the low BCECF fluorescence detected in P15–17 flowers (Fig. 2D) coincided with the moderate change in break strength.

**Fig. 3.**
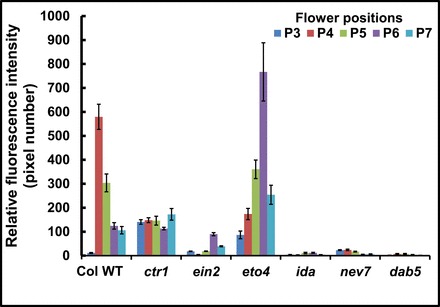
Relative fluorescence intensity quantified for the micrographs of BCECF images presented in Figs 1 and 2 of flower organ AZ of *Arabidopsis* Col WT and ethylene- and abscission-related mutants showing pH changes in P3–P7 flowers. The relative fluorescence intensity of flower organ AZ of the WT and the indicated mutants was quantified by confocal microscope MICA software. The data represent means of 3–4 replicates ±SE.

Quantification of the BCECF fluorescence in P3–P7 flowers in *Arabidopsis* WT and the mutants is presented in Fig. 3. The data confirm the pattern of changes presented in Figs 1 and 2, showing a decreased fluorescence in P4 and P7 flowers in the WT, a relatively moderate fluorescence in P3 in *ctr1*, a barely detected fluorescence in *ein2*, a marked increase in fluorescence in P3 and P6 flowers in *eto4*, and an almost undetectable fluorescence in *ida*, *nev7*, and *dab5*. In summary, the pattern of AZ-specific BCECF fluorescence correlates well with the abscission process in *Arabidopsis* WT and in both ethylene-dependent and -independent abscission mutants.

### A specific increase of the cytosolic pH in flower organ AZ cells coincided with flower organ abscission in control and ethylene- and 1-MCP-treated wild rocket flowers

**Fig. 4.**
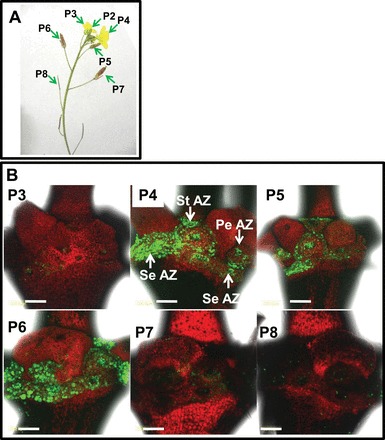
Flower developmental stages in wild rocket (*Diplotaxis tenuifolia*) according to flower position (P) on the shoot (A), and fluorescence micrographs of BCECF images of flower organ AZ (B) showing pH changes in P3–P8 flowers. The arrows in the P4 flower indicate the location of the flower organ AZ, based on a scanning electron micrograph of *Arabidopsis* flowers (Patterson, 2001). PeAZ, petal AZ; StAZ, stamen AZ; SeAZ, sepal AZ. Scale bar=200 μm. The BCECF fluorescence examination was performed as detailed in Fig. 1. The experiment was repeated twice with 2–4 different biological samples of different flowering shoots, and similar results were obtained.

Wild rocket belongs to the same family as *Arabidopsis*, the Brassicaceae. Wild rocket is useful for comparison, not only because it is a different genus of Brassicaceae, but also because its plants are larger and easier to work with. The inflorescence architecture of wild rocket is similar to that of *Arabidopsis*, exhibiting a gradient of flower development down the inflorescence (Fig. 4A), with P3, P4, P5, P6, P7, and P8 representing a freshly open flower, a fully open flower, a senescing flower, a flower with abscising petals, an early developed silique, and a developed silique in which the floral organs abscised, respectively. It was observed that BCECF fluorescence was detected only in the AZ of flower organs in P4–P6 flowers, in which abscission was in progress (Fig. 4B). In P3 flowers, which just opened, and in P7 or P8 flowers, in which the abscission of floral organs was complete, BCECF fluorescence was barely detected (Fig. 4B).

**Fig. 5.**
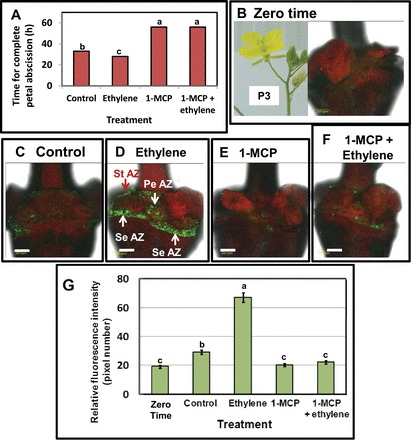
Effects of ethylene, 1-MCP, and a combined treatment of both on wild rocket petal abscission (A) and the expression of intracellular BCECF fluorescence in the AZ of P3 flower organs at zero time (B) and 24h after the initiation of the experiment (C–F), and on the level of the relative BCECF fluorescence intensity (G). The time for reaching complete petal abscission in response to the treatments was monitored in (A). For the fluorescence measurements, wild rocket inflorescences, in which P3 flowers were marked at zero time (B), were kept untreated at 20 °C for 24h as control (C), or exposed to ethylene (D), 1-MCP (E), or a combined treatment (F). Intact flowers were sampled from the inflorescences before or 24h after the ethylene/1-MCP treatments, incubated in BCECF solution, and examined by CLSM. The BCECF fluorescence analysis was performed as detailed in Fig. 1. The white arrows in (D) indicate the location of the flower organ AZs. StAZ, stamen AZ; PeAZ, petal AZ; SeAZ, sepal AZ. Scale bar=200 μm. The relative fluorescence intensity in (G) was quantified by confocal microscope MICA software, and the data represent means of four replicates ±SE. The results in (A) represent means of three biological experiments with 10 replicates each. Different letters above the bars in graphs A and G represent significant differences between treatments at *P*≤0.01.

To examine the effects of ethylene and/or 1-MCP on petal abscission of wild rocket flowers, cut inflorescences were used, in which the flower positions P0–P3 were marked before exposure to 10 μl l^–1^ ethylene for 24h. Flowers at positions P0–P3 responded to ethylene treatment, resulting in enhanced petal abscission; conversely, the combined treatment of 1-MCP and ethylene delayed petal abscission (data not shown). The effects of ethylene and 1-MCP on the timing of petal abscission in P3 flowers are presented in Fig. 5A, with ethylene accelerating abscission by 5h. However, in P0–P2 flowers the effect of ethylene on abscission was even more pronounced, accelerating abscission by 41, 29, or 17h in P0, P1, and P2 flowers, respectively (data not shown). Confocal fluorescent imaging of freshly open and non-abscising P3 flowers demonstrated that BCECF green fluorescence was barely detectable (Fig. 5B, G). After 24h, the intensity of the BCECF fluorescence, which increased slightly in the AZ of control flowers (Fig. 5C, G), significantly increased in the AZ of ethylene-treated flowers (Fig. 5D, G). Pre-treatment with 1-MCP inhibited the slight increase in fluorescence observed in control flowers after 24h (Fig. 5E, G), and completely abolished the ethylene-increased green fluorescence (Fig. 5F, G). These data indicate that the pH changes preceded the onset of petal abscission in both the control and ethylene-treated flowers. Thus, a moderate pH increase in the AZ cells of control P3 flowers was already observed 24h after the initiation of the experiment (Fig. 5C, G), before petal abscission was detected, whereas a complete petal abscission occurred only after 33h (Fig. 5A). Similarly, the ethylene-induced pH changes in the AZ cells of P3 flowers were observed 24h after the initiation of the experiment (Fig. 5D, G), while complete petal abscission in response to ethylene was obtained only after 28h (Fig. 5A). The results indicate that, similar to *Arabidopsis*, AZ-specific changes in pH occurred during abscission in wild rocket, and the changes in pH preceded the onset of organ abscission.

### 1-MCP blocked abscission and the increase in cytosolic pH in tomato flower AZ after flower removal

The kinetics of pedicel abscission in non-treated and 1-MCP-treated tomato inflorescence explants after flower removal was described previously (Meir *et al.*, 2010). Similar results were obtained in the present research (data not shown). Briefly, if tomato inflorescences, the panicle, were excised from the plant but the flowers remained attached, no pedicel abscission was observed during a 60h period following cluster detachment. Flower removal induced pedicel abscission within 10h, when ~15% of the pedicels abscised following a very slight touch. After 8h, no abscission was visible, but cell separation was already initiated. This indicates that the abscission process actually started earlier than 8h after flower removal. After 16h, 75% of the pedicels abscised. Pre-treatment with 1-MCP completely blocked pedicel abscission induced by flower removal for at least 20h after flower removal.

**Fig. 6.**
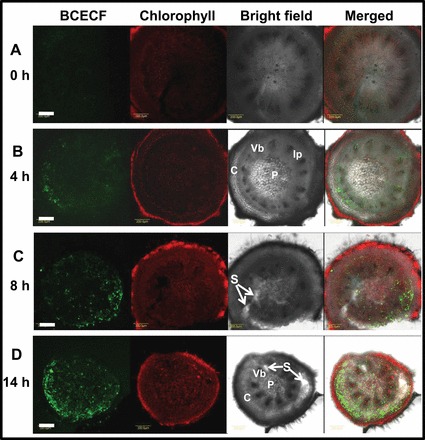
Fluorescence micrographs of BCECF, and chlorophyll autofluorescence, bright field, and merged images of cross-sections of the AZ of tomato flower pedicels showing pH changes at 0 (A), 4 (B), 8 (C), and 14 (D) h after flower removal. At the indicated time points after flower removal, cross-sections were made of the AZ of tomato flower explants held in water, incubated in BCECF solution, and examined by CLSM. Samples of zero time were excised from explants without flower removal. C, cortex; Vb, vascular bundles; Ip, interfascicular parenchyma; P, pith; S marked with arrows indicates regions in which cell separation already occurred. Scale bars=200 μm. The experiment was repeated twice with 3–4 different biological samples of different flowering shoots, and similar results were obtained.

The tomato FAZ is easily distinguished as a swollen node in the pedicel tissue (Roberts *et al.*, 1984; André *et al.*, 1999). In median cross-sections of the tomato FAZ, the BCECF green fluorescence appeared first in the swollen node 4h after flower removal, as a discrete peripheral spot of cells that included the vascular bundle and the surrounding parenchyma cells in the cortical side of the AZ (Fig. 6B). At 8h (Fig. 6C) and 14h (Fig. 6D) following flower removal, when separation occurred, the BCECF fluorescence was more intense and covered the entire cross-section. However, the most intense fluorescence appeared in the ring of cortical parenchyma cells between the vascular bundle and the epidermis (Fig. 6C, D). In the centre of the AZ node there is a region of relatively large parenchyma pith cells, which developed a weak fluorescence 14h after flower removal, just before abscission occurred. Nonetheless, the fluorescence intensity decreased 8h and 14h after flower removal in regions in which cell separation had already occurred and also in the vascular bundle (Fig. 6C, D). Magnification of the image in Fig. 6D, taken from parenchyma cells surrounding the vascular bundle 14h after flower removal (Supplementary Fig. S1C at *JXB* online), clearly shows that the intense fluorescence was located in the cytosol of the AZ of living cells, while the dead AZ cells (indicated by the white arrow in Supplementary Fig. S1C) displayed a much lower fluorescence, which appeared only in the vacuole. These results are in agreement with previous observations (Lampl *et al.*, 2013), showing that the BCECF fluorescence rapidly accumulated in the cytoplasm of the living epidermal cells, but when cells began to die the BCECF fluorescence was detected in the vacuole.

**Fig. 7.**
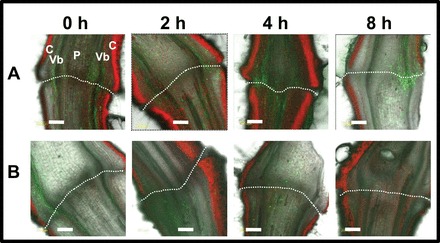
Fluorescence micrographs of BCECF images in longitudinal sections of tomato flower AZ showing pH changes in response to flower removal (A) and 1-MCP applied before flower removal (B) at the indicated time points after flower removal. Tomato flower explants held in water were exposed to 1-MCP (0.4 μl l^–1^ for 2h at 20 °C) prior to flower removal. Control flower explants were kept under similar conditions for the same period, and then flowers were removed. Samples of zero time were excised from explants without flower removal. At the indicated time points after flower removal, longitudinal sections of the AZ were prepared and incubated in BCECF solution as detailed in Fig. 1. C, cortex; Vb, vascular bundles; P, pith. The location of the AZ is indicated by a white dashed line. Scale bars=200 μm. The experiment was repeated twice with 3–5 different biological samples of different flowering shoots, and similar results were obtained.

Visualization of BCECF fluorescence in longitudinal sections of the FAZ displayed an increase in fluorescence in the vascular bundle and the cortex across the entire AZ (Fig. 7A). In this experiment, the fluorescence was observed in the FAZ at 0h. However, pre-treatment with 1-MCP, which completely abolished the tomato pedicel abscission for up to 38h after flower removal (Meir *et al.*, 2010), also completely abolished the increase in the BCECF fluorescence at all time points after flower removal (Fig. 7B). These results indicate that there is a correlation between pedicel abscission and alkalization of the cytosol in the tomato FAZ cells.

### Changes in the expression of genes that regulate cellular pH in tomato FAZ cells in response to flower removal and 1-MCP

A major regulatory mechanism of cellular pH is through the control of H^+^-related transport across membranes, including membrane transport of H^+^ between the cytosol and the two main acidic compartments, the apoplast and the vacuole. This is primarily facilitated by directly energized H^+^ pumps, including P-type H^+^-ATPase, V-type H^+^-ATPase, H^+^-pyrophosphatase (H^+^-PPase), and plant ion/H^+^ exchangers (Felle, 2005; Ortiz-Ramirez *et al.*, 2011; Pittman, 2012). Additional processes that could markedly affect cellular pH are nitrate and/or ammonium transporters and GTP-binding proteins (Lee and Yang, 2008; Bloch *et al*., 2011*a*, *b*; Luo *et al.*, 2013).

**Fig. 8.**
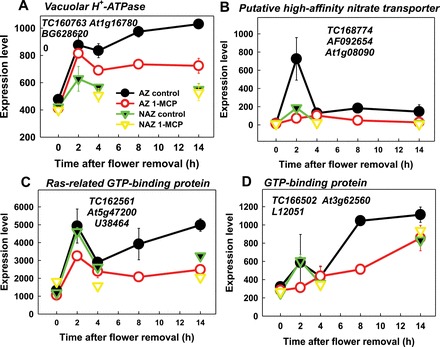
Effects of flower removal, 1-MCP pre-treatment, and tissue type on the kinetics of changes in array-measured expression levels of genes encoding pH regulatory transporters in tomato flower pedicels. Expression levels were measured for tomato *vacuolar H* ^*+*^ *-ATPase* (A), *putative high-affinity nitrate transporter* (B), *Ras-related GTP-binding protein* (C), and *GTP-binding protein* (D) transcripts. RNA samples were extracted from flower AZ or NAZ tissues taken from untreated (control) or 1-MCP-pre-treated tomato flower explants at the indicated time points after flower removal. The results are means of 2–3 biological replicates ±SD. Transcript identities are indicated by their tentative consensus sequence (TC) numbers in The Institute for Genomic Research (TIGR) and/or accession numbers. The microarray experiment was performed as described in Meir *et al.* (2010).

Microarray analysis of the abscission-related transcriptome in the tomato FAZ in response to auxin depletion revealed changes in expression of many genes occurring prior to and during pedicel abscission (Meir *et al.*, 2010). Some of these genes may be involved in the regulation of cellular pH, such as *vacuolar H* ^*+*^ *-ATPase* (BG628620), a gene encoding a putative high-affinity nitrate transporter (AF092654), and two genes encoding GTP-binding proteins (U38464 and L12051). Microarray analysis revealed an increase in expression of these four genes in the FAZ. Thus, v*acuolar H* ^*+*^ *-ATPase* (BG628620) expression increased by ~2-fold within 2h after flower removal and continued to increase slightly until 14h only in the AZ (Fig. 8A), indicating that it is AZ-specific. In 1-MCP-pre-treated flower clusters, the expression of this gene in the FAZ decreased after 2h and was significantly lower than that of the control (Fig. 8A). The expression of the high-affinity nitrate transporter gene (AF092654), which was transiently up-regulated specifically in the FAZ 2h after flower removal, was inhibited by 1-MCP pre-treatment (Fig. 8B). The two GTP-binding genes showed a transient increase in expression 2h after flower removal, which was not AZ-specific, followed by a more steady increase in expression between 4h and 14h, which was AZ-specific (Fig. 8C, D). The expression of both GTP-binding genes was inhibited or reduced by 1-MCP pre-treatment (Fig. 8C, D).

## Discussion

### The AZ-specific increase in pH coincides with the execution of natural organ abscission

It is well established that pH controls a variety of processes in plant cells, and might also serve as a signal for gene expression (Savchenko *et al.*, 2000; Felle, 2005, 2006; Couldwell *et al.*, 2009). Although it was hypothesized many years ago that pH changes might be involved in the abscission process (Osborne, 1989), this hypothesis was not experimentally tested and confirmed until now. The pH-sensitive BCECF dye exhibits an increase in green fluorescence at 488nm when the intracellular pH is in the range of pH 6.5–9 (Li *et al.*, 2008; Mumm *et al.*, 2011). Esterification of the carboxylic acid groups in BCECF with acetoxymethyl (AM) results in a non-fluorescent, uncharged molecule that can permeate cell membranes. Once inside the cell, the ester groups are cleaved by non-specific esterases, resulting in a fluorescent, charged BCECF molecule that is ion-trapped within the cell (Supplementary Fig. S1 at *JXB* online).

The concept of the AZ being a pre-determined site for specific inter- and intracellular signalling events is well established. There is convincing morphological, biochemical, and molecular evidence that cells which constitute the AZ respond to hormonal, developmental, and environmental cues differently from the neighbouring cells (Osborne, 1989; Roberts *et al.*, 2000 2002; Taylor and Whitelaw, 2001; González-Carranza *et al.*, 2002; Agusti *et al.*, 2009; Meir *et al.*, 2010). AZ cells, classified as type II ethylene-responsive target cells, exhibit a specific response to auxin and ethylene application as compared with NAZ cells, which are classified as type I cells (Osborne, 1982, 1989). The results presented herein show for the first time that pH changes are AZ-specific and coincide with the execution of abscission in three different abscission systems. The present data indicate a gradual specific increase in the cytosolic pH of AZ cells during natural abscission of flower organs in *Arabidopsis* (Fig. 1A) and wild rocket (Fig. 4B). A similar increase in pH was observed during pedicel abscission in tomato (Figs 6, 7), but the pH changes were less AZ-specific (Fig. 7A).

Abscission of *Arabidopsis* flower organs has been well characterized by using light and scanning microscopy and studies of AZ-specific GUS (β-glucuronidase) reporter gene expression, which included *PG*, *CHITINASE*, *HAE*, *EVERSHED*, and *BEAN ABSCISSION CELLULASE* (Bleecker and Patterson, 1997; González-Carranza *et al.*, 2002; Patterson and Bleecker, 2004; Butenko *et al.*, 2006; Liljegren *et al.*, 2009). The pattern of BCECF fluorescence, which indicates a change in pH in *Arabidopsis* P4–P7 flowers (Fig. 1A), was similar to the GUS staining pattern of the above AZ-specific genes. A similar AZ-specific fluorescence was observed in the AZ of wild rocket flower organs, which also coincided with cell separation (Fig. 4B). The tomato FAZ is typically composed of 5–10 rows of small cells, which traverse the pedicel at the site of an indentation of the epidermis. The FAZ cells, however, are not lined up, and there are regions that can contain >20 rows of cells (Rančić *et al.*, 2010; Iwai *et al.*, 2013). Nonetheless, the pattern of fluorescence changes during tomato flower pedicel abscission, as seen in cross- and longitudinal sections of the FAZ (Figs 6, 7), were similar to the pattern of GUS staining of the *Tomato Abscission PG4* (*TAPG4*) gene in cross- and longitudinal sections of the tomato FAZ following ethylene-induced abscission (Hong *et al.*, 2000). The similarity between *TAPG4::GUS* expression and BCECF fluorescence indicates that a specific pH increase in the AZ cells coincides in time and location with the AZ-specific *PG* expression that reflects execution of cell separation in the AZ.

### Ethylene induces abscission and increases the pH in AZ cells

To demonstrate a close correlation between ethylene-induced abscission and the alkalization of AZ cells, we used three experimental systems: ethylene-associated mutants of *Arabidopsis* (*ctr1*, *ein2*, and *eto4*), ethylene- and/or 1-MCP-treated wild rocket flowers, and 1-MCP-pre-treated tomato explants. The results obtained for these systems demonstrate a clear positive correlation between ethylene-induced abscission and an increase in the pH that is specific to the AZ cells.

The *ein2 Arabidopsis* mutant displays a delayed abscission phenotype (Patterson and Bleecker, 2004), but the abscission of *ctr1* and *eto4* mutants has not been well studied. In the *ein2* mutant, BCECF fluorescence was barely seen along the inflorescence (Fig. 1C), indicating that almost no change in pH occurred as compared with the WT. Conversely, the results presented in Supplementary Fig. S4 at *JXB* online show that floral organ abscission was significantly faster in *eto4*, as all floral organs in P5 flowers abscised, and alkalization in the AZ cells correlated with abscission (Figs 1D, 3). It was hypothesized that the enhanced abscission in *eto4* resulted from ethylene overproduction in the flowers. Monitoring ethylene production in flowers and siliques along the inflorescence of *eto4* in comparison with Col WT and the *ctr1* mutant indeed showed a significantly higher ethylene production rate in *eto4* P2–P7 flowers compared with the WT (Supplementary Fig. S6). On the other hand, the ethylene production rate in the siliques in *eto4* P10–P17 flowers was lower than that of the WT. It is interesting to note that the ethylene production rate in flowers and siliques along the inflorescence of the *ctr1* mutant was significantly lower than those of the WT in all flower stages (Supplementary Fig. S6).

Earlier studies indicated that in *eto1*, *2*, and *3* mutants, the post-transcriptional regulation of 1-aminocyclopropane-1-carboxylic acid (ACC) synthase (ACS) was affected (Woeste *et al.*, 1999; Chae *et al.*, 2003). Ethylene overproduction in the *eto1* and *3* mutants was limited mainly to etiolated seedlings, while light-grown seedlings and various adult tissues, including flowers, produced ethylene levels close to those of the WT (Woeste *et al.*, 1999). The *eto4* mutant, on the other hand, overproduced ethylene in P2–P5 flowers and P6–P7 young siliques of light-grown plants (Supplementary Fig. S6 at *JXB* online). However, the mechanism for overproduction of ethylene in *eto4* is unknown.

The floral organ abscission phenotype of *ctr1* is unique. In most ethylene-responsive systems examined, *ctr1* manifests itself as constitutively ethylene responsive (Keiber *et al*., 1993). One report was found regarding floral organ abscission in *ctr1*, which indicated that floral senescence/abscission in this mutant was similar to that of WT flowers (Chen *et al.*, 2011). The present results demonstrate that petals and sepals abscised earlier in the *ctr1* mutant, starting in the P5 flower (Supplementary Fig. S3 at *JXB* online); however, their abscission was incomplete, and some flower organs, mainly anthers, remained attached even in P9 flowers. The BCECF fluorescence in *ctr1* correlated with the abscission pattern, and a significant fluorescence intensity could be observed in P3 flowers (Figs 1B, 3), earlier than in the WT (Fig. 1A). The earlier abscission was not induced by ethylene, since the ethylene production rate in flowers and siliques along the inflorescence of *ctr1* was very low (Supplementary Fig. S6).

Exposure of *Arabidopsis* WT to ethylene enhances floral organ abscission (Butenko *et al.*, 2003). These authors observed that ethylene treatment (10 μl l^–1^ for 48h) of mature plants induced abscission in P1 flowers. Ethylene enhanced petal abscission of wild rocket, which started in P0–P3 flowers, while 1-MCP delayed it (Fig. 5A), suggesting that endogenous ethylene plays a role in wild rocket abscission. However, the floral organs of 1-MCP-treated flowers eventually abscised (Fig. 5A), indicating the involvement of an ethylene-independent abscission pathway in this species, similar to *Arabidopsis*.

As shown for *Arabidopsis*, ethylene treatment that enhanced flower petal abscission in wild rocket (Fig. 5A) significantly enhanced the increase in cytosolic pH, which was AZ-specific (Fig. 5D, G). Conversely, 1-MCP, which delayed petal abscission (Fig. 5A), completely inhibited the ethylene-induced pH increase after 24h (Fig. 5F, G). The pH changes preceded the onset of petal abscission (Fig. 5A) in both the control and ethylene-treated flowers (Fig. 5C, D, G), suggesting that they might be involved in the regulation of the abscission process. Similar to the results obtained with wild rocket, pre-treatment of tomato explants with 1-MCP, which inhibited pedicel abscission after flower removal (Meir *et al.*, 2010), also abolished the pH increase in the AZ cells (Fig. 7).

### pH changes in AZ cells of *Arabidopsis* flower organs also occur in an ethylene-independent abscission system

Since an ethylene-independent signalling pathway has been established in *Arabidopsis* floral organ abscission (Patterson and Bleecker, 2004), it was examined whether the AZ-specific pH changes also correlated with abscission in ethylene-independent mutants. Thus, in *Arabidopsis ida* and *nev7* mutants, in which floral organs do not abscise, the BCECF fluorescence was significantly lower than that detected in the WT (Figs 2B, C, 3). The low intensity of BCECF fluorescence in P4–P10 flowers in *ida* and in P3–P6 flowers in *nev7* does not contradict the general correlation between abscission and the increase in pH. In fact, these pH changes correlated well with the V-shape changes in the petal break strength in *ida* (ligand), *hae*/*hsl2* (receptors), and *nev7* (Butenko *et al.*, 2003; Cho *et al.*, 2008; Stenvik *et al.* 2008; Chen *et al.*, 2011; Liu *et al.*, 2013). The expression of hydrolytic and cell wall-modifying genes increased in flower stages 12–15 (Smyth *et al.*, 1990), although their expression was generally lower in *ida* and *hae*/*hsl2* than in the WT (Neiderhuth *et al*., 2013). The decrease in petal break strength suggests that cell wall-modifying enzymes and proteins are active in these mutants, but at a lower level than in the WT. Thus, the pH changes correlate well with the abscission-related changes in break strength and cell wall-modifying gene expression in these mutants, as well as in the WT and the ethylene-insensitive mutants (Figs 1–3). In addition, *dab5*, whose abscission starts only in P15 flowers (Supplementary Fig. S5 at *JXB* online), also showed BCECF fluorescence only in the AZ cells of P15–P17 flowers (Fig. 2D). However, this fluorescence was much lower than that of the WT (Fig. 2A) and *eto4* (Fig. 1D), whose abscission process occurred very rapidly. These results suggest that alkalization of the AZ cells is important for cell separation in both ethylene-dependent and -independent abscission processes.

### Changes in expression of genes that might regulate pH changes in AZ cells following abscission induction

The present results show a correlation between the increase in cytosolic pH and abscission in the AZ cells (Supplementary Fig. S1 at *JXB* online). A change in pH can affect many physiological processes and responses in plant cells (Savchenko *et al.*, 2000). The increase in intracellular pH in the AZ cells might be regarded as a component of the signal transduction pathway, leading to acquisition of abscission competence, and might serve in turn as a signal for abscission-related gene expression. In addition, alkalization of the cytosol might be reflected in the acidification of the apoplast, as apoplast acidification involves H^+^ extrusion from the cytoplasm by H^+^-ATPases and specific transporters (Grignon and Sentenac, 1991). The acidification of the apoplast might activate cell wall-modifying enzymes (Osborne, 1989). Indeed, it was recently reported that when ethephon-treated leaf petioles of *Phaseolus vulgaris* were subjected to pH 3.5 or 5.5, which altered the apoplast pH, abscission occurred, whereas at pH 7 abscission was inhibited (Fukuda *et al.*, 2013). However, these authors obtained opposite results in roots of *Azolla filiculoides*, in which a decrease in pH inhibited abscission. The authors suggest that the striking difference in pH sensitivity between *A. filiculoides* and *P. vulgaris* might be ascribed to a different pH optimum of pectin-degrading enzymes in these species.

Here, it was clearly demonstrated that intracellular alkalization correlates with abscission, but it is also important to determine how the increase in pH occurs. In this regard, microarray results might provide clues for the regulation of pH in the AZ cells. One possible mechanism could be via modified expression of AZ-specific transporter genes, such as *vacuolar-type H* ^*+*^ *-translocating ATPase*, *plasma membrane H* ^*+*^ *-ATPase*, *nitrate and/or ammonium transporter*, and *GTP-binding proteins* (Fig. 8). All of the above gene families that might regulate pH changes showed AZ-specific expression changes during organ abscission in microarray analyses of various abscission systems, such as *Arabidopsis* stamens (Cai and Lashbrook, 2008), citrus leaves (Agusti *et al.*, 2009), apple flowers (Zhu *et al.*, 2011), mature fruits of olive (Gil-Amado and Gomez-Jimenez, 2013) and melon (Corbacho *et al.*, 2013), and tomato flower pedicels (Meir *et al.*, 2010; Wang *et al.*, 2013). In the tomato flower pedicel system (Wang *et al.*, 2013) and citrus leaves (Agusti *et al.*, 2009), abscission was induced by exogenous ethylene, but in all the other systems the abscission was dependent on endogenous ethylene. Thus, the transcriptome data clearly show that ethylene-dependent changes in expression of many genes are involved in abscission regulation and execution, including genes encoding proteins that regulate the pH in AZ cells. ATPases and membrane transporters could be regulated post-transcriptionally by a variety of signals; but some might be regulated transcriptionally. To verify this possibility, earlier microarray results (Meir *et al.*, 2010) were examined for changes in H^+^-translocating ATPases, nitrate and/or ammonium transporters, and GTP-binding proteins. Four genes were discovered in the FAZ whose expression increased during abscission in an AZ-specific manner and was inhibited by 1-MCP treatment (Fig. 8). The role of these genes in abscission remains to be studied. It is interesting to note that recently the orthologues of these genes were expressed in *Arabidopsis* floral organ AZ, and the gene encoding the high-affinity nitrate transporter (At1g08090, Fig. 8B) significantly increased in flower stages 12–15, in which floral organ abscission occurred (Neiderhuth *et al*., 2013). An additional support for these findings was recently reported, showing that several genes encoding various H^+^-ATPases, ammonium transporter, and Rab GTP-binding were up-regulated during ethylene-induced tomato flower abscission (Wang *et al.*, 2013). Taken together, the above data provide further evidence for the involvement of pH changes in the process of organ abscission, which might be regulated via specific modification of transporters in AZ cells.

### Conclusions

The present novel results demonstrate that AZ-specific pH changes occur in the cytosol of AZ cells, which are induced by both ethylene-sensitive and -insensitive signalling pathways. These changes coincide with the execution of floral organ abscission following abscission induction in all the examined systems, as well as with the decreased break strength in *Arabidopsis*. pH can affect enzymatic activities and/or act as a signal for gene expression. Therefore, the results open a new and challenging direction for abscission research.

## Supplementary data

Supplementary data are available at *JXB* online.

Figure S1. Fluorescence micrographs of BCECF images of flower organ AZ of *Arabidopsis* Col WT in P5 flower and of a cross-section of tomato flower pedicel AZ excised 14h after flower removal, showing a high intensity of green fluorescence in the cytosol.

Figure S2. Abscission phenotypes of flowers and siliques in P3–P8 flowers of *Arabidopsis* Col WT.

Figure S3. Abscission phenotypes of flowers and siliques in P1–P10 flowers of *Arabidopsis ctr1* mutant.

Figure S4. Abscission phenotypes of flowers and siliques in P1–P6 flowers and in four representative replicates of the upper inflorescences of the *Arabidopsis eto4* mutant.

Figure S5. Abscission phenotypes of flowers and siliques in P3–P16 flowers of the *Arabidopsis dab5* mutant.

Figure S6. Ethylene production rates in P2–P17 flowers and siliques of *Arabidopsis* Col WT and *ctr1* and *eto4* mutants.

Supplementary Data

## Abbreviatons:

AZ: abscission zone
BCECF-AM: 2’,7’-bis-(2-carboxyethyl)-5(and-6)-carboxy-fluorescein-acetoxymethyl
CLSM: confocal laser scanning microscope
COI1: CORONATINE INSENSITIVE 1
ctr1: constitutive triple response 1
DAB: delayed in abscission
DDW: double distilled water
DMSO: dimethyl sulphoxide
ein2: ethylene-insensitive 2
eto4: ethylene overproducer 4
etr1: ethylene receptor 1
FAZ: flower abscission zone
HAE: HAESA
HSL2: HAESA-LIKE2
IDA: INFLORESCENCE DEFICIENT IN ABSCISSION
1-MCP: 1-methylcyclopropene
NAZ: non-abscission zone
NEV: nevershed
PBS: phosphate-buffered saline
PG: polygalacturonase
TAPG4: Tomato Abscission PG4
WT: wild type.
