# Supplementary material for: Abscission of flowers and floral organs is closely associated with alkalization of the cytosol in abscission zone cells
Source: J Exp Bot. 2014 Dec 10;66(5):1355–68. doi: 10.1093/jxb/eru483 (PMC4339595; doi:10.1093/jxb/eru483)
Supplement: Supplementary Data [file supp_66_5_1355__index.html]

Abscission of flowers and floral organs is closely associated with alkalization of the cytosol in abscission zone cells — Abscission of flowers and floral organs is closely associated with alkalization of the cytosol in abscission zone cells — Supplementary Data 

# Abscission of flowers and floral organs is closely associated with alkalization of the cytosol in abscission zone cells

## Supplementary Data

Data files

**Files in this Data Supplement:**

- Supplementary Data - Supplementary Data
