# Supplementary material for: Abscission of flowers and floral organs is closely associated with alkalization of the cytosol in abscission zone cells
Source: J Exp Bot. 2014 Dec 10;66(5):1355–68. doi: 10.1093/jxb/eru483 (PMC4339595; doi:10.1093/jxb/eru483)
Supplement: Supplementary Data [file supp_eru483_jexbot131276_file001.pdf]

Supplementary data

Abscission of flowers and floral organs is closely associated with alkalization of the cytosol in the abscission zone cells

Srivignesh Sundaresan, Sonia Philosoph-Hadas, Joseph Riov, Eduard Belausov, Bettina Kochanek, Mark L. Tucker and Shimon Meir

Supplementary Fig. S1.

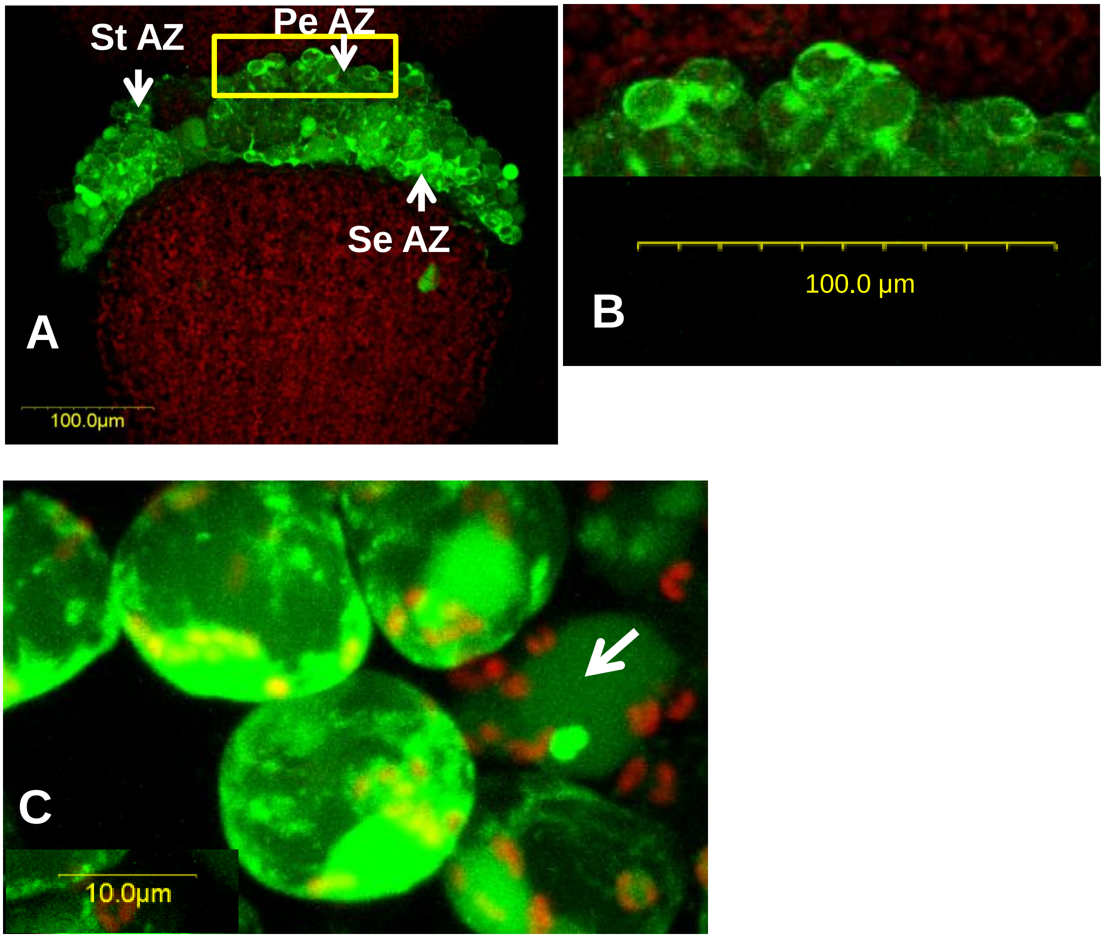

Figure S1. Fluorescence micrographs of BCECF images of flower organ AZs of Arabidopsis Col WT at position P5 (A, B) and of a cross section of tomato flower pedicels AZ taken 14 h after flower removal (C), showing a high intensity of green fluorescence in the cytosol. At the indicated flower position for Arabidopsis or time point after flower removal for tomato, samples were incubated in BCECF solution and examined by CLSM. The image in (B) represents a magnification of the Arabidopsis petal AZ cells indicated by the yellow rectangular in (A). The image in (C) shows living parenchyma cells surrounding the vascular bundles in the tomato flower pedicel AZ, in which the BCECF dye accumulated in the cytosol at a high intensity, and a dead cell (indicated by the white arrow), in which the BCECF dye accumulated in the vacuole at a low intensity. PeAZ, Petal AZ; StAZ, stamen AZ; SeAZ, sepal AZ. Scale bars = 100 μm (A, B) and 10 μm (C).

Supplementary Fig. S2.

WT Col

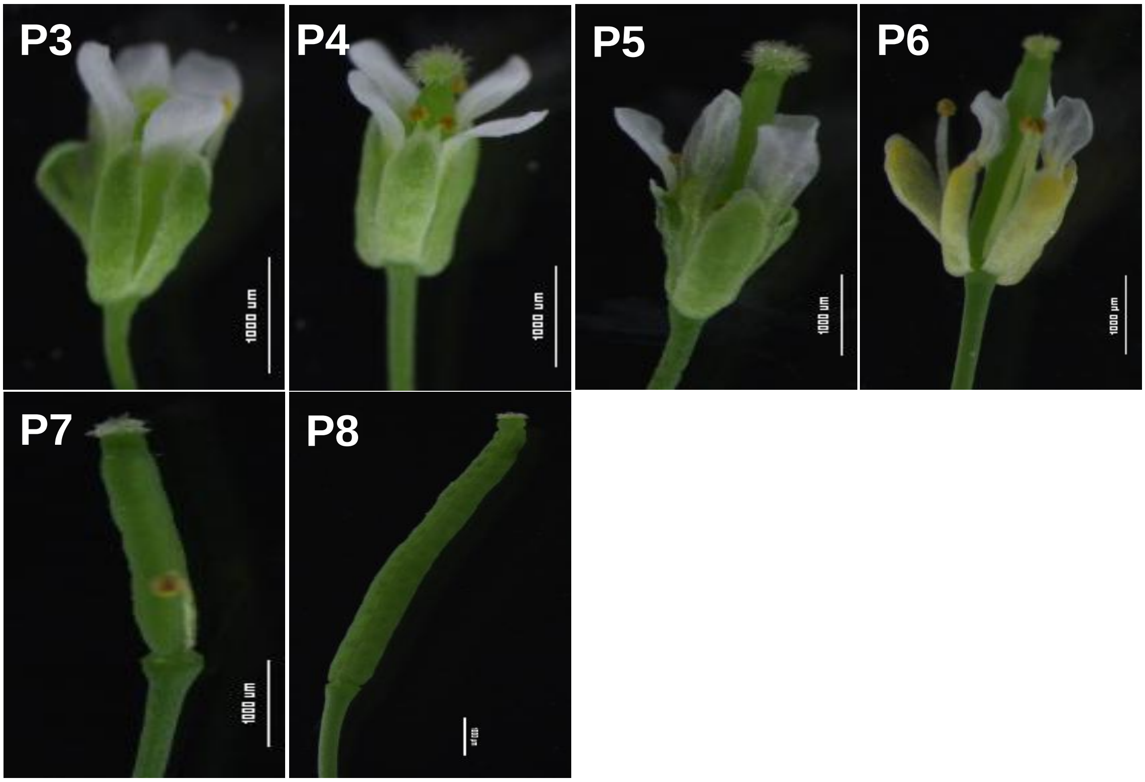

Figure S2. Abscission phenotypes of flowers and siliques in P3 to P8 flowers of Arabidopsis Col WT. P3 represents a fully open flower, P5 a withering flower, P6 a senescent flower, and P7 a flower in which all the petals abscised. Scale bars = 1000 µm.

Supplementary Fig. S3.

ctr1

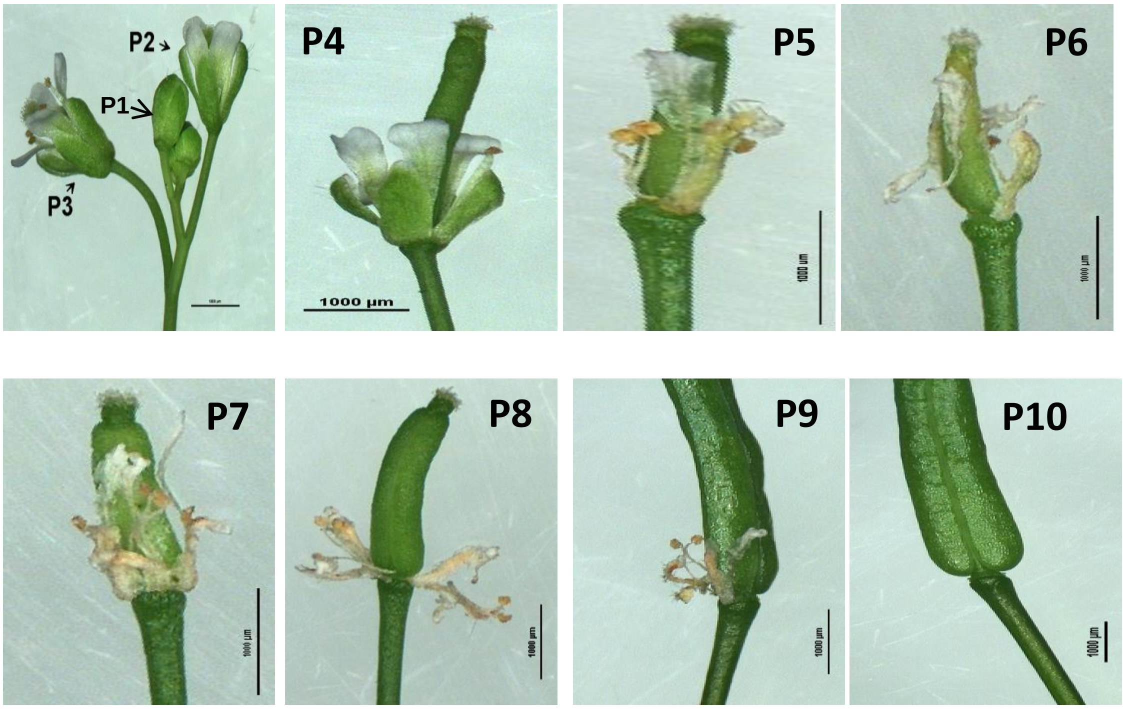

Figure S3. Abscission phenotypes of flowers and siliques in P1 to P10 flowers of Arabidopsis *ctr1* mutant. P1 represents the stage in which flower petals are first visible, and P3 a fully open flower (indicated by arrows). Scale bars = 1000 µm.

Supplementary Fig. S4.

eto4

A *eto4* flower Position

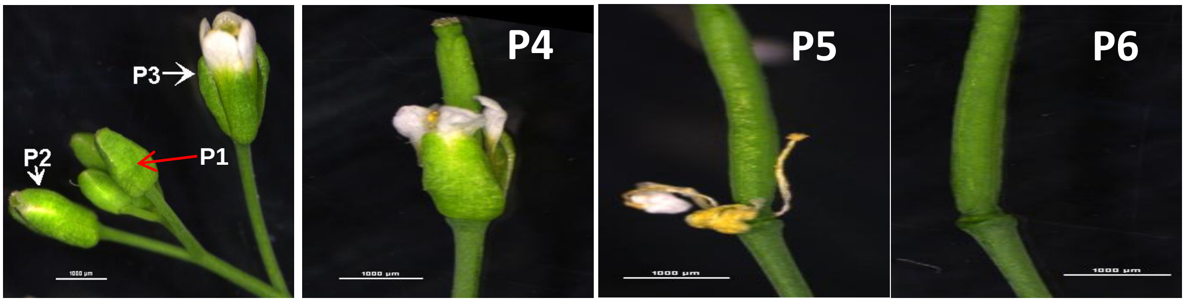

B *eto4* inflorescences

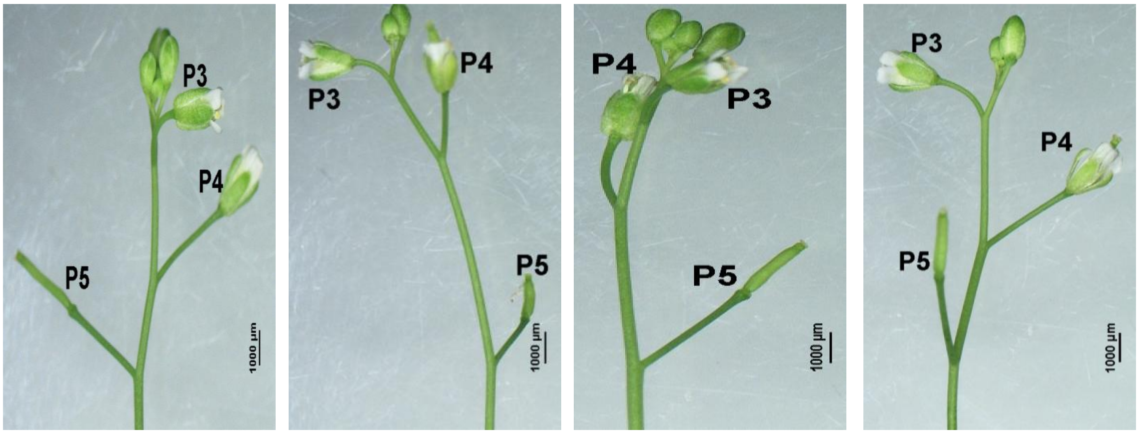

Plant 1 Plant 2 Plant 3 Plant 4

Figure S4. Abscission phenotypes of flowers and siliques in P1 to P6 flowers (A) and in four representative replicates of the upper inflorescences (B) of Arabidopsis *eto4* mutant. P1 represents the stage in which flower petals are first visible, P3 a fully open flower, and P5 a flower in which all the petals abscised or some residues remained attached. Scale bars = 1000 μm.

Supplementary Fig. S5.

dab5

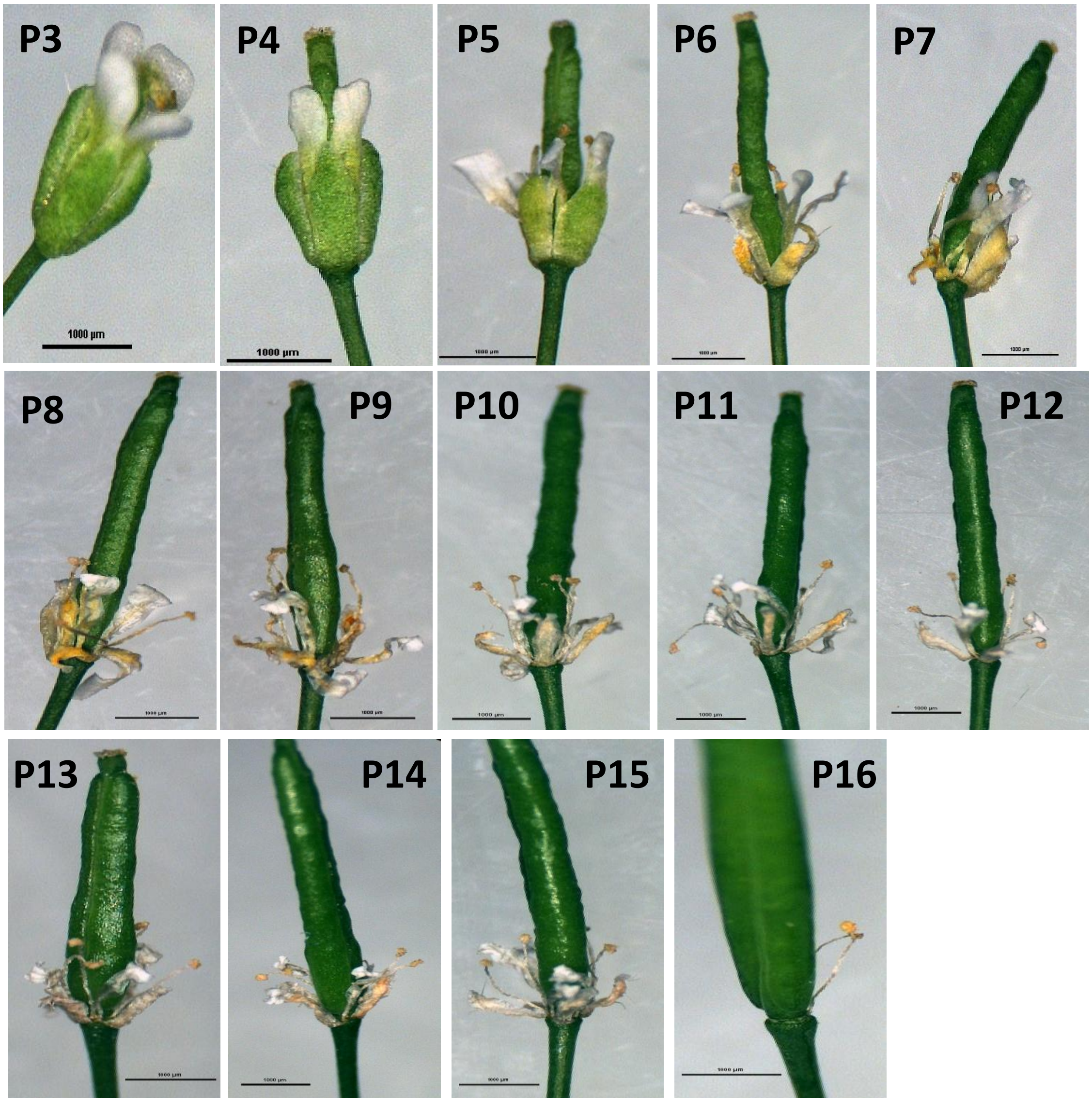

Figure S5. Abscission phenotypes of flowers and siliques in P3 to P16 flowers of Arabidopsis *dab5* mutant. P3 represents a fully open flower, P5 a withering flower, and P6 to P15 senescent flowers which do not abscise. Scale bars = 1000 µm.

Supplementary Fig. S6.

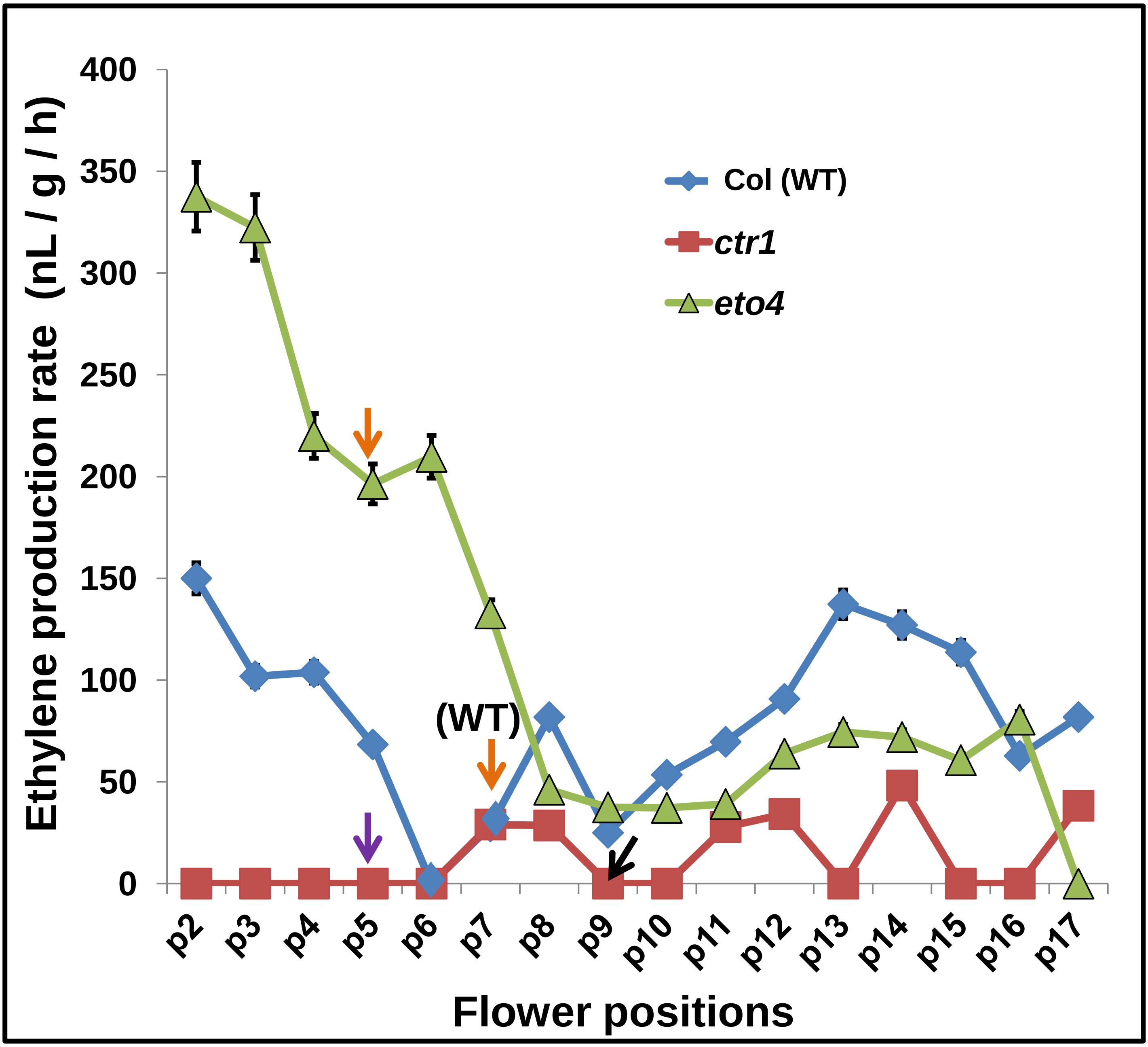

Figure S6. Ethylene production rates of flowers and siliques at positions P2 to P17 of Arabidopsis Col WT and *ctr1* and *eto4* mutants. The data represent means ± SE of two replicates, each containing 7-8 flowers and/or siliques. Initiation of abscission of flower organs is indicated by colored arrows when separation was observed. A purple arrow represents initiation of petals and sepal abscission, a black arrow represents initiation of stamen abscission, and the orange arrows represent initiation of abscission of all flower organs.
